# Deleterious Mutation Accumulation in *Arabidopsis thaliana* Pollen Genes: A Role for a Recent Relaxation of Selection

**DOI:** 10.1093/gbe/evz127

**Published:** 2019-06-18

**Authors:** Mark C Harrison, Eamonn B Mallon, Dave Twell, Robert L Hammond

**Affiliations:** Department of Genetics and Genome Biology, University of Leicester, United Kingdom

**Keywords:** purifying selection, sporophyte, pollen, ploidy, deleterious, masking

## Abstract

In many studies, sex-related genes have been found to evolve rapidly. We therefore expect plant pollen genes to evolve faster than sporophytic genes. In addition, pollen genes are expressed as haploids which can itself facilitate rapid evolution because recessive advantageous and deleterious alleles are not masked by dominant alleles. However, this mechanism is less straightforward to apply in the model plant species *Arabidopsis thaliana*. For 1 Myr, *A. thaliana* has been self-compatible, a life history switch that has caused: a reduction in pollen competition, increased homozygosity, and a dilution of masking in diploid expressed, sporophytic genes. In this study, we have investigated the relative strength of selection on pollen genes compared with sporophytic genes in *A. thaliana*. We present two major findings: 1) before becoming self-compatible, positive selection was stronger on pollen genes than sporophytic genes for *A. thaliana* and 2) current polymorphism data indicate that selection is weaker on pollen genes compared with sporophytic genes. This weaker selection on pollen genes can in part be explained by their higher tissue specificity, which in outbreeding plants can be outweighed by the effects of haploid expression and pollen competition. These results indicate that since *A. thaliana* has become self-compatible, selection on pollen genes has become more relaxed. This has led to higher polymorphism levels and a higher build-up of deleterious mutations in pollen genes compared with sporophytic genes.

## Introduction

A faster evolution of reproductive genes compared with somatic genes has been documented for a wide range of taxa, including primates, rodents, mollusks, insects, and fungi (Swanson and Vacquier 2002; Turner and Hoekstra 2008). The faster evolution is often observable in a higher number of nonsynonymous nucleotide substitutions (base changes which alter the amino acid sequence of a protein) within the coding regions of orthologs. In most cases, stronger positive selection is described as the mechanism driving the divergence of these genes, generally due to some form of sexual selection like cryptic female choice or sperm competition (Swanson and Vacquier 2002).

Three studies on the strength of selection on reproductive and nonreproductive genes in *Arabidopsis thaliana* have presented somewhat conflicting findings (Szövényi et al. 2013; Gossmann et al. 2014, 2016). Gossmann et al. (2014), who were primarily interested in the effect of sex-biased expression, found no difference in protein divergence (d*N*/d*S*; ratio of nonsynonymous to synonymous per site substitution rates) between male genes and 476 random, nonreproductive genes sampled from the *A. thaliana* genome. Female-biased genes, on the other hand, show significantly higher d*N*/d*S* rates. Interestingly, in a more recent study, when analyzing the full transcriptome, pollen genes were found to be evolving faster than sporophytic genes (Gossmann et al. 2016), indicating the gene selection method may play a crucial role. Szövényi et al. (2013), who compared evolutionary rates between haploid and diploid stages, showed that the rate of protein evolution (d*N*/d*S*) between *A.thaliana* and *Arabidopsislyrata* of pollen-specific genes was significantly higher than for sporophyte-specific genes (Szövényi et al. 2013). The additional detection, in the Szövényi et al. study, of higher intraspecific polymorphism levels within pollen genes was compatible with relaxed purifying selection on pollen genes. This is because stronger positive selection, that could have caused the higher divergence rates, would have reduced intraspecific polymorphism levels. High tissue specificity and higher expression noise compared with sporophytic genes were considered the likely causes of relaxed selection on pollen genes (Szövényi et al. 2013). Expression noise describes the stochasticity of expression level between individuals and is thought to negatively affect the efficacy of selection in a similar manner to a reduction in population size (Wang and Zhang 2011). As pointed out by Gossmann et al. (2014), which focused on the comparison of genes with male biased or female-biased expression, interspecific divergence and currently existing intraspecific polymorphisms likely arose under different selection regimes for *A. thaliana*. The divergence of *A. thaliana* from its closest relative *A. lyrata* happened largely during a period of outcrossing, since speciation occurred ∼13 Ma (Beilstein et al. 2010), whereas *A. thaliana* became self-compatible only roughly 1 Ma (Tang et al. 2007). Divergence patterns for *A. thaliana* should therefore be similar to outcrossing species and reveal stronger selection on pollen genes. Existing, intraspecific polymorphisms, on the other hand, are expected to be influenced by high selfing rates in *A. thaliana* populations that have led to high levels of homozygosity across the whole genome (Nordborg 2000; Wright et al. 2008; Platt et al. 2010). The outcome is a reduction in the masking of deleterious alleles in diploid sporophyte stages (because of high homozygosity) compared with the haploid gametophyte stage. Furthermore, selfing will result in fewer genotypes competing for fertilization so lowering the magnitude of pollen competition and reducing the strength of selection acting on pollen (Charlesworth D and Charlesworth B 1992).

Despite using a larger number of accessions to measure polymorphism than in the Szövényi et al. study (80 compared with 19), Gossmann et al. did not detect any difference in nucleotide diversity between the nonreproductive genes and pollen-specific genes in general, although nucleotide diversity was significantly lower for sperm cell-specific genes (Gossmann et al. 2014). When comparing polymorphism to divergence data with a modified version of the McDonald–Kreitman test (McDonald and Kreitman 1991, Distribution of Fitness Effects Software, DoFE; Eyre-Walker and Keightley 2009), in contrast to the Szövényi et al. study, a higher proportion of nonsynonymous sites were found to be under purifying and adaptive selection for pollen genes compared with both female-biased and nonreproductive genes.

The aim of our study was to attempt to resolve these apparently conflicting results for *A. thaliana* and to address the following questions. Are pollen proteins really more divergent than sporophyte proteins? If so, is this due to more relaxed purifying selection or increased positive selection on pollen genes, or both? Have patterns of selection changed for *A. thaliana* since it became self-compatible? In a first step, we estimated the protein divergence of 1,552 pollen and 5,494 sporophytic genes to both *A. lyrata* and *Capsella rubella* in terms of interspecific d*N*/d*S*. We estimated the proportion of sites under positive and negative selection within the two groups of genes by conducting a DoFE analysis. As the polymorphism and divergence data likely reflect periods of differing selection regimes (divergence under largely self-incompatibility, polymorphism under self-compatibility), we additionally detected sites under positive selection using a site model of the Phylogenetic Analysis by Maximum Likelihood software (PAML 4.6; Yang 2007), which does not require polymorphism data and detects sites under positive selection by allowing d*N*/d*S* to vary within genes. In a second step, to investigate more recent selection patterns, we analyzed intraspecific polymorphism levels within each group of genes. Lower diversity, measured here via nonsynonymous Watterson’s *θ* and nucleotide diversity (*π*) (when controlling for synonymous diversity), would be expected for pollen genes compared with sporophyte genes in the case of stronger selection (Nielsen 2005). In a further test, we also compared existing levels of putative deleterious alleles (premature stop codons and frameshift mutations) between pollen genes and sporophyte genes. In each of these analyses, we controlled for differences in genomic factors (expression level, GC content, codon bias, gene density, gene length, and average intron length) between the pollen and sporophyte-specific genes which were correlated with divergence, polymorphism, and deleterious allele measurements.

## Materials and Methods

### Genomic Data

Publicly available variation data were obtained for 269 inbred strains of *A. thaliana*. Beside the reference genome of the Columbia strain (Col-0), which was released in 2000 (*Arabidopsis*, Genome Initiative), 250 were obtained from the 1001 genomes data center (http://1001genomes.org/datacenter/; accessed September 2013), 170 of which were sequenced by the Salk Institute (Schmitz et al. 2013) and 80 at the Max Planck Institute, Tübingen (Cao et al. 2011). A further 18 were downloaded from the 19 genomes project (http://mus.well.ox.ac.uk/; accessed September 2013; Gan et al. 2011). These 269 files contained information on single nucleotide polymorphisms (SNPs) and indels recorded for separate inbred strains compared with the reference genome. A quality filter was applied to all files, in order to retain only SNPs and indels with a phred score of at least 25. For further analyses, gene sequences were created for each of these strains based on coding sequence information contained in the TAIR10 gff3 file.

### Expression Data

Normalized microarray data, covering 20,839 genes specific to different developmental stages and tissues of *A. thaliana* (supplementary table S1, Supplementary Material online), were obtained from Borg et al. (2011). The expression data consisted of seven pollen and ten sporophyte data sets (supplementary table S1, Supplementary Material online). Four of the pollen data sets represented expression patterns of the pollen developmental stages, uninucleate, bicellular, tricellular, and mature pollen grain, one contained expression data of sperm cells and the remaining two were pollen tube data sets. There was a strong, significant correlation between the two pollen tube data sets (*ρ *= 0.976; *P* < 2.2 × 10^−^^16^; Spearman’s rank correlation), so both were combined and the highest expression value of the two sets was used for each gene. Each of the ten sporophyte data sets contained expression data for specific sporophytic tissues (supplementary table S1, Supplementary Material online).

Each expression data point consisted of a normalized expression level (ranging from 0 to around 20,000, scalable and linear across all data points and data sets) and a presence score ranging from 0 to 1 based on its reliability of detection across repeats, as calculated by the MAS5.0 algorithm (Borg et al. 2011). In our analyses, expression levels were conservatively considered as present if they had a presence score of at least 0.9, whereas all other values were regarded as zero expression.

Genes were classed as either pollen- or sporophyte-specific genes, if expression was reliably detectable in only pollen or only sporophyte tissues or developmental stages. The highest expression value across all tissues or developmental stages was used to define the expression level of a particular gene. The highest value was used since this best represents the genes’ most important effect on the phenotype. We also consider tissue specificity of expression to fully explain a gene’s expression profile.

### Detecting Signatures of Selection

#### Evolutionary Rates

To estimate evolutionary rates of genes, d*N*/d*S* ratios (ratio of nonsynonymous to synonymous substitution rates relative to the number of corresponding nonsynonymous and synonymous sites) were calculated for all orthologous genes between pairs of the three species *A. thaliana*, *A. lyrata*, and *C.rubella* using the codeml program within the PAML package (Yang 2007). The protocol described in Szövényi et al. (2013) was followed. Orthologs were found by performing reciprocal BlastP searches (Altschul et al. 1997) between proteomes and retaining protein pairs with mutual best hits showing at least 30% identity along 150 aligned amino acids (Rost 1999). Orthologous protein sequences were aligned with MUSCLE (Edgar 2004) at default settings and mRNA alignments were performed based on these protein alignments with pal2nal (Suyama et al. 2006). The codeml program was run with runmode -2, model 2 and “NSsites” set to 0. Genes with a d*S* > 2 were removed from the analysis. In most results, we report divergence (d*N*/d*S*) between *A. thaliana* and *A. lyrata* unless otherwise stated.

In order to detect genes that contain codon sites under positive selection, we performed a likelihood-ratio test (LRT) between models 7 (null hypothesis; d*N*/d*S* limited between 0 and 1) and 8 (alternative hypothesis; additional parameter allows d*N*/d*S* > 1) by using runmode 0, model 0 and setting “NSsites” to 7 and 8. An LRT statistic (twice the difference in log-likelihood between the two models) >9.210 indicated a highly significant difference (*P* < 0.01; LRT > 5.991: *P* < 0.05) between the two models suggesting the existence of sites under positive selection within the tested gene (Anisimova et al. 2003; Yang 2007). These tests were carried out on multispecies alignments containing orthologs from *A. thaliana*, *A. lyrata*, and *C. rubella* that were contained in each of the three ortholog lists described before. Alignments were carried out in the same manner as described above for pairs of sequences.

Levels of purifying and positive selection were estimated by calculating the distribution of fitness effects (DFEs) using grapes (Galtier 2016) and implementing the Gamma0 model developed by Eyre-Walker and Keightley (2009). For the input files, polymorphism data were extracted from the genome matrix for the 80 accessions of the Cao et al. (2011) release, only retaining sites for which a base was reliably sequenced. Four-fold sites were used to represent synonymous positions and 0-fold degenerate sites to represent nonsynonymous positions. Four-fold and 0-fold sites were calculated with perl scripts; any codons containing more than one SNP were removed from the analysis. We compiled input files for the DFE analysis with custom made scripts for all sporophyte and pollen genes, for which divergence data and polymorphism data from all 80 strains were available. Values were summed across all genes. To generate 95% confidence intervals, we resampled with replacement and reran grapes 1,000 times.

#### Intraspecific Polymorphism

Nucleotide diversity (*π*) and Watterson’s *θ* were calculated for nonsynonymous sites using the R package PopGenome (version 2.1.6; Pfeifer et al. 2014). The diversity.stats() command was implemented and the subsites option was set to “nonsyn.” Both values were subsequently divided by the number of sites.

#### Putatively Deleterious Alleles

To quantify the frequency of deleterious mutations for each gene, the occurrence of premature stop codons and frameshifts was calculated for each gene locus across all 268 strains compared with the reference genome. Stop codons were recorded as the number of unique alternative alleles occurring within the 269 strains as a result of a premature stop codon. Frameshifts were calculated as a proportion of the strains containing a frameshift mutation for a particular gene. All analyses of coding regions were based on the representative splice models of the *A. thaliana* genes (TAIR10 genome release, www.arabidopsis.org).

#### Statistical Analyses

All analyses were performed in R (version 3.2.0; R Core Team 2012). To measure statistical difference between groups, we utilized the nonparametric Mann–Whitney *U* test (wilcox.test() function). In case of multiple testing, all *P* values were corrected with the Bonferroni method using the function p.adjust(). For correlations, either the Spearman rank test (rcorr() function of Hmisc package; version 3.16-0; Harrell and Dupont 2015) or Spearman rank partial correlation (pcor.test() function; ppcor package; version 1.0; Kim 2012) was carried out.

Six genomic parameters were investigated as possible predictors of d*N*/d*S*, polymorphism levels and frequency of deleterious mutations. These were expression level, GC content, codon bias variance, gene density, average gene length, and average intron length. Expression level is described above in the section “Expression data.” Average gene length and average intron length were calculated using custom made scripts which extracted information from the genomic gff file. GC content was calculated with a downloaded Perl script, which was originally written by Dr Xiaodong Bai (http://www.oardc.ohio-state.edu/tomato/HCS806/GC-script.txt). Relative synonymous codon usage was used to measure codon bias. It was calculated for each codon of each locus with the R package “seqinr” (uco() function; version 3.1-3; Delphine et al. 2014). As the mean value per gene varied very little between loci but varied by site within genes, we used relative synonymous codon usage variance as a measure for codon bias. Gene density was calculated with custom Perl and R scripts by counting the number of genes within each block of 100 kb along each chromosome. Gene densities were then attributed to each gene depending on the 100-kb window, in which they were situated.

As most of the genomic parameters investigated here (gene expression, GC content, codon bias variance, gene density, average gene length, and average intron length) generally differed between groups of genes (see Results), it was important to control for their possible influence on divergence, polymorphism, and frequencies of deleterious mutations. The six parameters were also intercorrelated, so we decided to implement principle component (PC) regression analyses (pcr() command, pls package, version 2.4-3; Mevik and Wehrens 2007) in order to combine these parameters into independent predictors of the variation in the investigated dependent variable (e.g., d*N*/d*S*) as described by Drummond et al. (2006). All variables, including the dependent variable, were log transformed (0.0001 was added to gene length and average intron length due to zero values). A jack knife test (jack.test()) was subsequently performed on each set of principal component regression results to test if the contribution of each predictor was significant. Nonsignificant predictors were then removed and the analyses were repeated. The PC, which explained the highest amount of variation in the dependent variable, was then used to represent the genomic predictors in an ANCOVA (e.g., lm[log{d*N*/d*S*} ∼ PC1 × ploidy]) with life-stage as the binary covariate.

## Results

### Life-Stage Limited Genes

Within the total data set, containing 20,839 genes, 4,304 (20.7%) had no reliably detectable expression (score < 0.9; see Materials and Methods) in any of the analyzed tissues and were removed from the analysis. Of the remaining 16,535 genes, 1,552 genes (9.4%) were expressed only in pollen and a further 5,494 (33.2%) were limited to sporophytic tissues (referred to as pollen-specific genes and sporophyte-specific genes in this study). The pollen-specific and sporophyte-specific genes were randomly distributed among the five chromosomes (table 1), and their distributions within the chromosomes did not differ significantly from each other (supplementary table S2, Supplementary Material online).

**Table 1:** Chi-Square Test of the Distribution of Pollen and Sporophyte Limited Genes among the Five Nuclear *Arabidopsis thaliana* Chromosomes

Chromosome	All Genes	Pollen	Sporophyte
1	4,348	392	1,495
2	2,522	251	862
3	3,326	340	1,049
4	2,451	214	839
5	3,888	355	1,249
Σ	16,535	1,552	5,494
	*χ* ^2^	5.367	7.456
	*P*	0.252	0.136

Note.—Degrees of freedom: 4.

Expression level was roughly twice as high within pollen-specific genes (median: 1,236.1) compared with sporophyte-specific genes (median: 654.7; *W* = 5.5 × 10^6^; *P* = 1.2 × 10^−^^63^; table 2, supplementary fig. S1, Supplementary Material online). GC content was significantly higher within sporophyte-specific genes (median: 44.6%) than in pollen-specific genes (median: 43.8%; *W* = 3.4 × 10^6^; *P* = 1.0 × 10^−^^19^; table 2, supplementary fig. S1, Supplementary Material online). Sporophyte-specific genes were significantly longer and contained significantly longer introns than pollen-specific genes (table 2 and supplementary fig. S1, Supplementary Material online). Pollen-specific genes were situated in regions of slightly but significantly higher gene density; codon bias variance did not differ significantly (table 2 and supplementary fig. S1, Supplementary Material online).

**Table 2:** Differences in Six Genomic Variables between Pollen-Specific and Sporophyte-Specific Genes

Genomic Variable	Pollen-Specific Genes		Sporophyte-Specific Genes	*P*
Expression level	2,562.30 ± 86.49	>	1,256.21 ± 23.80	1.2 × 10^−63^
GC content (%)	44.20 ± 0.08	<	45.08 ± 0.04	1.0 × 10^−19^
Codon bias variance	0.46 ± 0.01	=	0.43 ± 0.00	Not significant
Gene length	1,570.30 ± 24.41	<	1,634.39 ± 11.62	2.3 × 10^−4^
Average intron length	124.44 ± 3.23	<	160.08 ± 2.49	8.6 × 10^−10^
Gene density (per 100 kb)	29.99 ± 0.12	>	29.57 ± 0.07	1.5 × 10^−3^

Note.—Values are means ± standard error of the mean; significance was tested with Mann–Whitney *U* test; *P* values are Bonferroni corrected for multiple testing.

### Pollen-Specific Proteins Evolve at a Faster Rate than Sporophyte-Specific Proteins

**Fig. 1.**
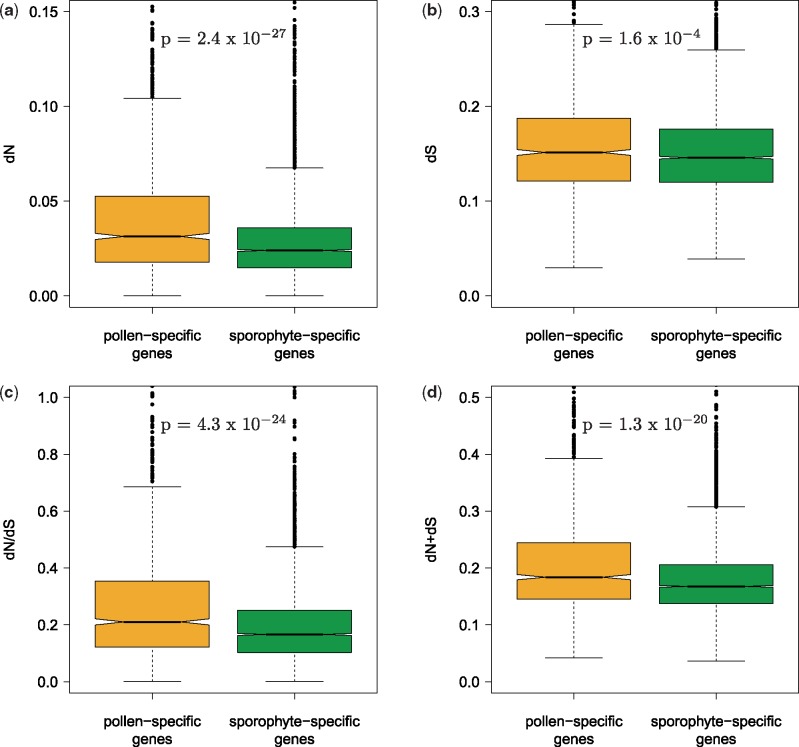
—Nonsynonymous (d*N*; *a*), synonymous (d*S*; *b*), d*N*/d*S* (*c*), and total nucleotide substitution rate (d*N* + d*S*; *d*) within pollen-specific and sporophyte-specific genes. Significance tested with Mann–Whitney *U* test.

The rate of evolution of *A.thaliana* proteins from *A.lyrata* orthologs was estimated using interspecific d*N*/d*S*. Of the 13,518 genes for which 1-to-1 orthology could be detected and d*N*/d*S* could be reliably analyzed, 1,144 genes were pollen specific and 4,395 were sporophyte specific. Protein divergence was significantly higher for pollen-specific genes (median: 0.208) than sporophyte-specific genes (median: 0.164; *P* = 4.3 × 10^−^^24^; fig. 1*c*). This was mainly due to a significant difference in the nonsynonymous substitution rate (d*N*) for which the median was 30.8% higher in pollen-specific genes (medians: 0.030 and 0.024; *P* = 2.4 × 10^−^^27^; fig. 1a). Synonymous divergence (d*S*) was only 3.7% higher in pollen-specific genes and the difference was less significant (medians: 0.148 and 0.145; *P* = 1.6 × 10^−^^4^; fig. 1*b*).

Both expression level (*ρ* = −0.232; *P* = 5.6 × 10^−^^169^) and GC content (*ρ* = −0.145; *P* = 4.3 × 10^−^^64^) were significantly negatively correlated with d*N*/d*S* while controlling for other factors (codon bias variance, gene length, average intron length, and gene density; table 3). Codon bias variance and gene length correlated weakly and negatively with d*N*/d*S*, whereas average intron length and gene density showed minimal correlation (table 3).

**Table 3:** Partial Correlations of Six Genomic Variables with d*N*/d*S*, *θ*_n_, *π*_n_, Frequency of Premature Stop Codons, and Frameshift Mutations

	d*N*/d*S*	*θ*_n_	*π*_n_	Stop Codons	Frameshifts
Expression level	−0.232***	−0.131***	−0.086***	Not significant	−0.090***
GC content (%)	−0.145***	−0.192***	−0.166***	−0.180***	−0.143***
Codon bias variance	−0.104***	−0.210***	−0.161***	−0.124***	−0.088***
Gene length	−0.108***	0.325***	0.181***	0.136***	−0.037*
Average intron length	−0.061***	−0.191***	−0.123***	0.084***	−0.109***
Gene density (per 100 kb)	0.039*	−0.137***	−0.116***	−0.054***	−0.029*

Note.—Spearman rank correlations controlling for remaining five variables; *P* values are Bonferroni corrected for multiple testing.

* *P* < 0.01; **P < 10^−6^; ***P < 10^−9^.

**Fig. 2.**
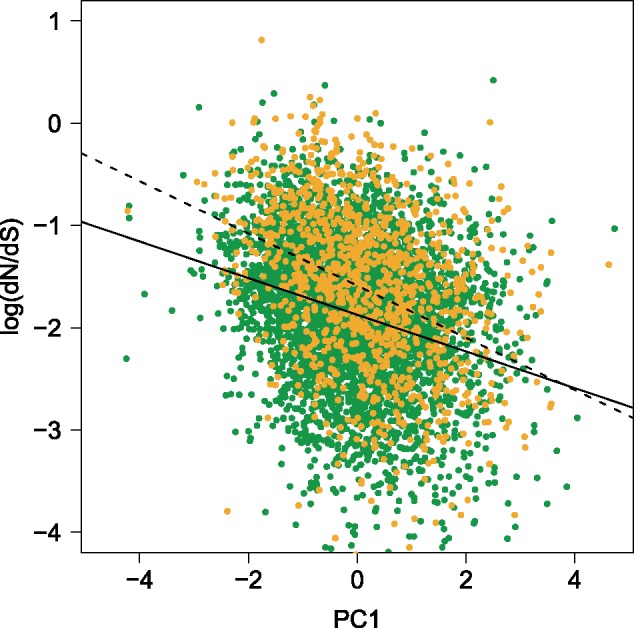
—ANCOVA of d*N*/d*S* within pollen-specific (yellow points and dashed line) sporophyte-specific genes (green points and solid line) with PC1 (expression and GC content) as the continuous variable.

In order to determine how the life-stage (pollen or sporophytic tissue), to which the expression of a gene is limited, may be contributing to the measured difference in d*N*/d*S*, it was important to control for the six previously mentioned genomic variables (expression level, GC content, codon bias variance, gene length, average intron length, and gene density). This was important because five of the six genomic variables differed significantly between pollen and sporophyte-specific genes (table 2) and all six were significantly correlated to d*N*/d*S* (table 3). A principal component regression was conducted to allow us to condense these predictors of d*N*/d*S* into independent variables. We first included all six predictors in the principal component regression model, and they explained 9.10% of d*N*/d*S* variation. Principal component (PC) 2 explained the largest amount of variation at 6.15%. A jack knife test on this PC revealed significant *P* values (<0.05) only for expression, GC content, and codon bias variance. After removal of the nonsignificant predictors (gene length, average intron length, and gene density), codon bias variance was also no longer significant. The first PC of a model containing expression and GC content as the predictors of d*N*/d*S* had an explanation value of 7.15% (total 7.24%). This first PC was used as the continuous variable in an ANCOVA with d*N*/d*S* as the dependent variable and life-stage as the binary covariable. The pollen regression line was higher than for the sporophyte genes for the majority of the PC1 range (fig. 2). As the slopes differed significantly (*P* = 4.4 × 10^−^^4^), we measured the difference in d*N*/d*S* between pollen and sporophyte genes within five equal bins along the PC1 axis. In all five quantiles, d*N*/d*S* was higher within pollen genes than within sporophyte-specific genes (highly significant in the first three, marginally significant in the fourth after correction and nonsignificant in the fifth quantile; table 4).

**Table 4:** d*N*/d*S* within Five Equal Bins along the PC1 Axis

	<20%	20–40%	40–60%	60–80%	>80%
Pollen	0.269 (0.347)	0.249 (0.346)	0.210 (0.276)	0.173 (0.214)	0.144 (0.198)
Sporophyte	0.220 (0.247)	0.173 (0.199)	0.160 (0.183)	0.146 (0.192)	0.132 (0.160)
*P*	3.7 × 10^−5^	1.4 × 10^−9^	1.6 × 10^−6^	0.050	Nonsignificant

Note.—Values are given as medians (means).

### Sporophyte-Specific Genes Contain a Higher Number of Sites under Purifying Selection

We investigated whether the higher divergence of pollen-specific proteins compared with sporophyte-specific proteins was restricted to *Arabidopis*, and possibly fueled by selection in either *A. thaliana* or *A. lyrata*, by investigating the protein divergence of both from *C.rubella*. Divergence was significantly higher for pollen-specific proteins in all three comparisons (supplementary table S3, Supplementary Material online).

**Fig. 3.**
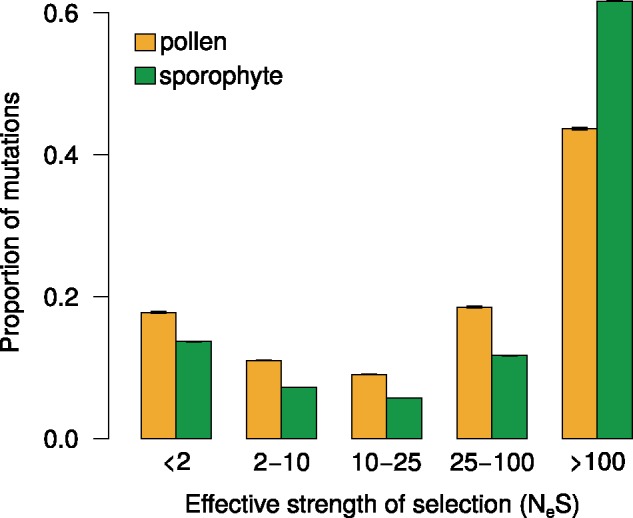
—DFE for pollen and sporophyte-specific genes. Shown are the mean proportions of mutations in four *N*_e_*s* ranges with 95% confidence intervals.

A higher d*N*/d*S* value, which is still lower than 1, generally indicates weaker purifying selection (Yang and Bielawski 2000). Only 41 out of 13,518 genes had a d*N*/d*S* value >1 and 65.1% of genes had a d*N*/d*S* < 0.2. However, gene-wide estimates of d*N*/d*S* can be inflated by a few codon sites under positive selection (>1) even if purifying selection is otherwise prevalent. In order to test whether the higher d*N*/d*S* within pollen genes was being driven by relaxed purifying selection or increased positive selection, we analyzed the DFEs of new mutations, using the grapes software and implementing the GammaZero model (Eyre-Walker and Keightley 2009; Galtier 2016). This was carried out on all genes, for which full information was available from all 80 accessions (Cao et al. 2011): 522 pollen genes and 2,039 sporophyte genes. The distribution of new deleterious mutations showed that a smaller fraction of nonsynonymous mutations were strongly deleterious (*N*_e_*s* > 10; *N*_e_: effective population size, *s*: selection coefficient) within pollen-specific genes (mean 0.713, 95% CI [0.709, 0.715]) compared with sporophyte genes (0.791, [0.790, 0.792]; fig. 3). Also, a higher proportion of mutations in pollen genes were effectively neutral (*N*_e_*s* < 2; 0.177, [0.177, 0.179]) compared with sporophyte genes (0.137, [0.136, 0.137]). This indicates weaker purifying selection within the pollen-specific genes (Eyre-Walker and Keightley 2009) and suggests the higher d*N*/d*S* rates in pollen genes may be caused by an accumulation of slightly deleterious mutations due to random drift.

Using the same software, we found a higher proportion of sites to be under positive selection (*α*) in sporophyte genes (0.327, [0.325, 0.329]) compared with pollen genes (0.319, [0.315, 0.322]). As estimations of *α* can be overestimated due to fluctuations in *N*_e_ (Rousselle et al. 2018), as can be expected for *A. thaliana* due to the recent emergence of self-compatibility, we conducted a further analysis to investigate levels of positive selection, which does not rely on polymorphism data. On a multisequence alignment containing single orthologs from each of the three species, *A. thaliana*, *A. lyrata*, and *C. rubella*, we allowed d*N*/d*S* to vary among sites in order to detect sites under positive selection using codeml in PAML (Yang 2007). This analysis, suggested a much higher proportion of pollen-specific genes contained sites under positive selection (15.2% at *P* < 0.05; 9.1% at *P* < 0.01) compared with sporophyte-specific genes (9.3% *P* < 0.05; 4.8% at *P* < 0.01). As expected, d*N*/d*S* was significantly higher within the genes containing sites under positive selection compared with genes with no evidence for positive selection (median of 0.338 compared with 0.179 for pollen genes, *P* = 3.8 × 10^−^^21^; 0.228 compared with 0.154 in sporophyte genes, 3.9 × 10^−^^24^). It appears, therefore, that at least a part of the difference in d*N*/d*S* is caused by a higher rate of adaptive fixations in pollen genes.

### Pollen-Specific Genes Are More Polymorphic than Sporophyte-Specific Genes

**Fig. 4.**
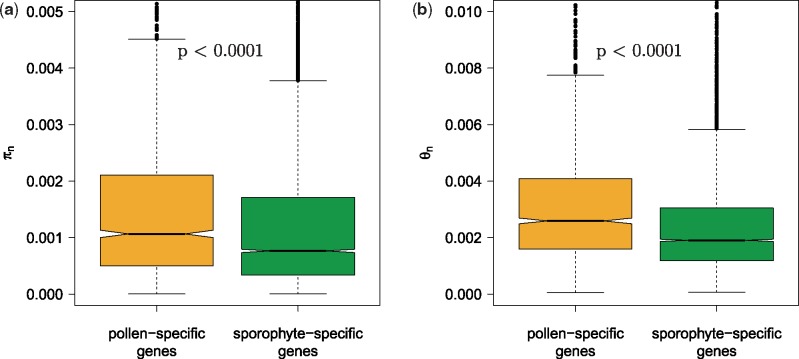
—Nonsynonymous nucleotide diversity (*a*) and nonsynonymous Watterson’s theta (*b*) within pollen-specific and sporophyte-specific genes. Significance tested with Mann–Whitney *U* test.

Pollen-specific genes were more polymorphic than sporophyte-specific genes with both nonsynonymous nucleotide diversity (*π*_n_) and nonsynonymous Watterson’s theta (*θ*_n_) significantly higher in pollen-specific genes (fig. 4). Both *π* and *θ* at synonymous sites did not differ between sporophyte- and pollen-specific genes (*P* = 0.18 and 0.58, respectively). Each of the six correlates of d*N*/d*S* listed above also correlated significantly with *π*_n_ and *θ*_n_ (all negatively except gene length; table 3). Five of the six variables (average intron length was not significant) explained 8.57% of variation in *π*_n_ in a principal component regression. The first PC contributed most (3.11%). Four of the six factors (expression level, GC content, codon bias variance, and gene density) explained a total of 7.76% of the variation in *θ*_n_ (first PC: 7.38%). For each model, the first PC was implemented in an ANCOVA testing the influence of life-stage as a covariate. *θ*_n_ remained significantly higher for pollen-specific genes (*P* = 6.4 × 10^−^^61^; fig. 2*b*). PC1 had a significantly greater influence on *π*_n_ for sporophyte genes (slope: −0.195) than on pollen genes (slope: −0.109; *P* = 7.2 × 10^−^^4^; fig. 2*a*). We therefore tested the significance of difference in *π*_n_ within five equal bins along the PC1 axis. In the second to the fifth 20% quantiles, *π*_n_ was significantly higher within pollen genes, there was no difference in the first quantile (supplementary table S4, Supplementary Material online).

### Higher Frequency of Deleterious Mutations in Pollen-Specific Genes

**Fig. 5.**
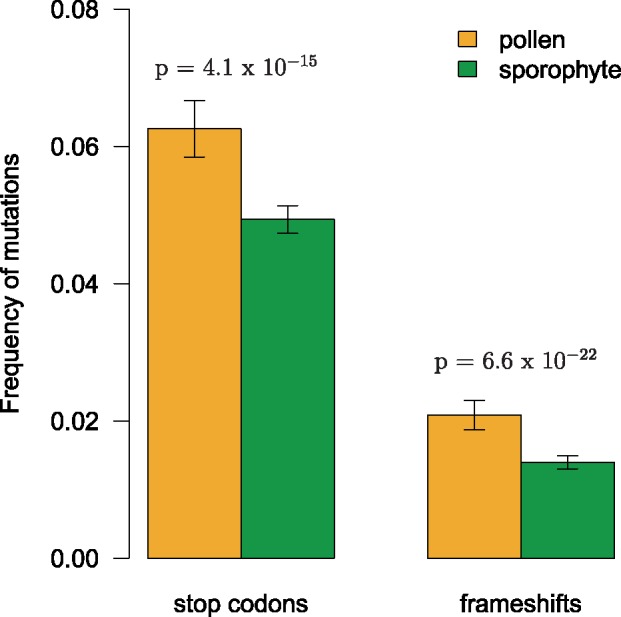
—Frequency of alleles containing premature stop codon mutations and frameshift mutations in pollen-specific and sporophyte-specific genes. Shown are means and standard error. Significance tested with Mann–Whitney *U* test.

Higher polymorphism levels may indicate relaxed purifying selection on pollen-specific genes. To test this hypothesis further, we investigated the frequency of putatively deleterious mutations—premature stop codons and frameshift mutations—within the 269 *A. thaliana* strains. Stop codon frequency, defined here as the relative number of unique alternative alleles due to premature stop codons occurring within the 269 strains, was significantly higher within pollen-specific genes (mean: 0.063 ± 0.004; sporophyte mean: 0.049 ± 0.002; *P* = 4.1 × 10^−^^15^; Mann–Whitney *U* test; fig. 5). The frequency of strains containing at least one frameshift mutation was also significantly higher for pollen-specific genes (mean: 0.021 ± 0.002) compared with sporophyte-specific genes (mean: 0.014 ± 0.001; *P* = 6.6 × 10^−^^22^; fig. 5). Significant correlations existed between these measures of deleterious mutations and the six correlates of d*N*/d*S* (table 3).

In a principal component regression analysis, all six predictors (expression level, codon bias variance, GC content, gene length, average intron length, and gene density) were significantly correlated with stop codon frequency. The six predictors explained a total of 20.04% of the variation in stop codon frequency, 17.42% explained by the first PC. Within an ANCOVA with life-stage as the binary covariant, the frequency of premature stop codons remained higher within pollen-specific genes for the majority of PC1 (fig. 3*a*). The slopes differed significantly but the frequency of stop codons was significantly higher for pollen genes within the second to fifth 20% quantiles (supplementary table S5, Supplementary Material online).

Four of the predictors (expression level, GC content, gene length, and gene density) were also significantly correlated with the frequency of frameshift mutations. However, the four variables only explained a total of 5.49% of variation (first PC 5.08%). In an ANCOVA, frameshift mutations remained significantly more frequent within pollen-specific genes when controlling for the predictors via the first PC (fig. 3*b*).

### Tissue-Specific Genes

Tissue specificity has been shown to be negatively correlated with selection efficiency (Duret and Mouchiroud 2000; Liao et al. 2006; Slotte et al. 2011). The on average greater tissue specificity in pollen-specific genes compared with sporophyte-specific genes could therefore potentially explain the higher polymorphism levels and higher frequency of deleterious mutations found in pollen-specific genes. In order to control for this potential bias, we compared d*N*/d*S*, polymorphism levels, and the frequency of deleterious alleles in pollen-specific genes with a group of 340 genes with expression limited to a single sporophyte cell type (guard cell, xylem, or root hair). To further test for the effect of tissue specificity, these groups were also compared against 2,543 genes which were expressed in at least five sporophytic tissues.

**Fig. 6.**
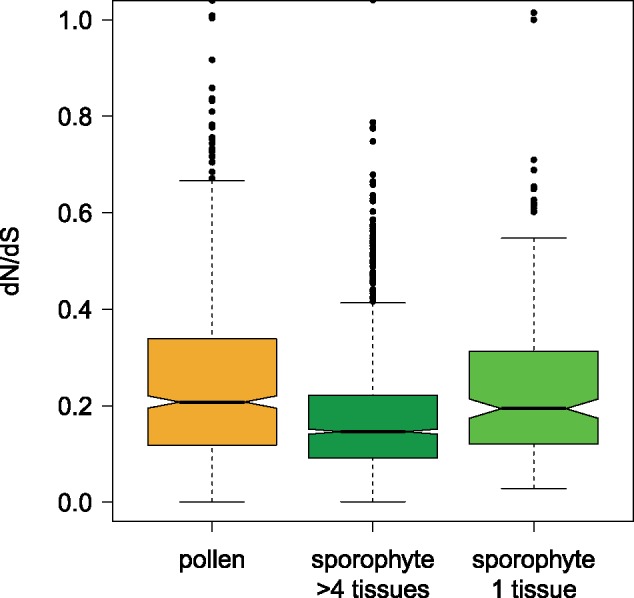
—d*N*/d*S* within pollen-specific genes, broadly expressed sporophytic genes (at least five tissues) and tissue-specific genes (expression restricted to guard cell, xylem, or root hair tissues).

In this tissue specificity controlled comparison, d*N*/d*S* did not differ between pollen-specific and the tissue-specific sporophyte gene set. However, d*N*/d*S* was significantly higher in pollen-specific genes (*P* = 1.7 × 10^−^^27^) and tissue-specific sporophyte genes (*P* = 1.0 × 10^−^^9^; fig. 6) compared with broadly expressed sporophyte-specific genes. In a principal components regression, only expression level and GC content had a significant effect on d*N*/d*S*, explaining 8.63% of variation. The PC1 (8.60%) was then mapped against d*N*/d*S* in an ANCOVA on the two levels pollen-specific genes and tissue-specific, sporophytic genes. d*N*/d*S* was significantly higher for pollen genes than tissue-specific, sporophytic genes when controlling for PC1 (*P* = 1.4 × 10^−^^3^; supplementary fig. S4, Supplementary Material online).

Similarly, *π*_n_ and *θ*_n_ did not differ between pollen-specific and the tissue-specific sporophyte gene set. However, they were both significantly higher in pollen-specific genes (*P* = 1.6 × 10^−^^30^ and 8.4 × 10^−^^75^, respectively) and tissue-specific sporophyte genes (*P* = 7.1 × 10^−^^13^ and 2.7 × 10^−^^26^; supplementary fig. S5, Supplementary Material online) compared with broadly expressed sporophyte-specific genes. In a principal components regression, expression level and GC content had a significant effect on *π*_n_, explaining 5.30% of variation. The first PC (5.06%) was plotted against *π*_n_ in an ANCOVA within pollen-specific and tissue-specific sporophytic genes. *π*_n_ was significantly higher for pollen-specific genes compared with tissue-specific, sporophytic genes when controlling for PC1 (*P* = 6.5 × 10^−^^3^; fig. 6*a*). In a similar analysis for *θ*_n_, all six parameters (expression level, GC content, codon bias variance, gene length, average intron length, and gene density) significantly contributed to 18.82% of variation. The second PC was largest (9.55%) and was plotted against *θ*_n_ in an ANCOVA (fig. 6*b*). When controlling for PC2 *θ*_n_ was significantly higher within pollen-specific genes (*P* = 2.9 × 10^−^^7^) compared with tissue-specific, sporophytic genes.

Premature stop codons remained significantly more frequent in pollen-specific genes than in sporophytic, tissue-specific genes (*P* = 0.033), and broadly expressed, sporophytic genes (*P* = 3.0 × 10^−^^14^; supplementary fig. S7, Supplementary Material online). Premature stop codons were more frequent in tissue-specific, sporophytic tissues (mean 0.057 ± 6.9 × 10^−^^3^) compared with broadly expressed sporophytic genes (0.051 ± 3.2 × 10^−^^3^) but not significantly. There was no significant difference in the frequency of frameshift mutations between pollen-specific genes and tissue-specific, sporophytic genes but the frequency was significantly higher in both groups compared with broadly expressed, sporophytic genes (*P* = 2.0 × 10^−^^34^ and 1.7 × 10^−^^14^; supplementary fig. S7, Supplementary Material online). GC content, codon bias variance, gene length, average intron length, and gene density had a significant effect on 19.03% variation in the frequency of stop codons. The first PC was largest (16.44%) and was implemented in an ANCOVA as the continuous variable (supplementary fig. 8*a*, Supplementary Material online). Due to a significant interaction between the two groups, pollen-specific genes and tissue-specific, sporophytic genes, differences in stop codon frequencies were measured within five equal bins along the PC1 axis. The frequency of stop codon mutations did not differ significantly within the first four quantiles but was significantly higher within pollen-specific genes in the fifth quantile (PC1 > 1.14; *P* = 5.7 × 10^−^^5^). The analysis was repeated for the frequency of frameshift mutations. Expression level, GC content, codon bias variance, and gene length explained a total of 6.35% variation. PC2 was largest with 3.24% so was implemented in an ANCOVA. The frequency of frameshift mutations was significantly higher within pollen-specific genes compared with tissue-specific, sporophytic genes when controlling for PC2 (*P* = 0.017; supplementary fig. 8*b*, Supplementary Material online).

## Discussion

Our analysis showed that protein divergence, polymorphism levels, and the frequency of deleterious mutations were significantly higher within pollen-specific genes compared with sporophyte-specific genes. These differences remained when controlling for expression level, GC content, codon bias variance, gene length, average intron length, and gene density.

### Evolutionary Rates Higher within Pollen-Specific Genes

Protein divergence rates (d*N*/d*S*) were on average 37% higher in pollen-specific genes compared with sporophyte-specific genes. This is comparable to the findings presented by Szövényi et al. (2013), who found d*N*/d*S* to be 39% or 81% higher in pollen genes for *A. thaliana* depending on the data set. In a further article, no difference in d*N*/d*S* could be found between pollen-specific and nonreproductive genes for *A. thaliana* (Gossmann et al. 2014) (table 5). This discrepancy was most likely caused by the method of gene selection. In the Szövényi et al. (2013) study, as in the current study, genes with exclusive expression within sporophytic or pollen tissues were analyzed. In the Gossmann et al. (2014) article, on the other hand, genes were selected more inclusively, labeling a gene as pollen-enriched if expression was significantly higher at a fold change >4 within different comparisons. This means that at least some of the sporophyte genes discussed in the Gossmann et al. (2014) study will also be expressed to some extent within pollen tissues and are therefore exposed to haploid selection. Even a low level of expression in haploid tissues may be sufficient to counteract the effect of masking, which would explain the lack of difference in evolutionary rates detected. The importance of gene selection was further confirmed by Gossmann et al. (2016) who found more rapid evolution in pollen tissues when considering the entire transcriptome. It appears then that the genes, which are exclusively expressed in pollen or sporophytic tissues, may be causing the significantly different d*N*/d*S* rates we observe here.

**Table 5:** Comparison of Selection Analyses on Pollen and Sporophytic Genes in *Arabidopsis thaliana* from This and Two Previous Studies

Study	Data Set	Results
Szövényi et al. (2013)	Number of genes in two data sets: 64 and 425 haploid-specific genes, 2,598 and 2,699 diploid-specific genes, 8,806 and 8,246 unspecific genes. Polymorphism data: 19 strains	d*N*/d*S*: haploid > diploid > unspecific. p*N*/p*S*: haploid > diploid > unspecific
Gossmann et al. (2014)	14,159 pollen genes, 11,657 pollen tube genes, 7,832 sperm cell genes, and 476 random genes.^a^ Polymorphism data: 80 strains	d*N*/d*S*: no difference between pollen and random genes. Nucleotide diversity: no difference between pollen and random genes, lower in sperm cell genes. DFE: greater purifying and adaptive selection in pollen genes (excluding sperm cells) than in random genes
Current study	1,552 pollen-specific genes and 5,494 sporophyte-specific genes. Polymorphism data: 80 strains for DFE analysis, otherwise 269	d*N*/d*S*: pollen genes > sporophyte genes. *π*_n_ and *θ*_n_: pollen genes > sporophyte genes. Stop codons and frameshifts: pollen genes > sporophyte genes. DFE: weaker purifying selection in pollen genes, stronger adaptive selection in sporophyte genes. Positive selection based on divergence data: pollen > sporophyte genes

^a^This study also analyzed genes with expression bias in female tissues.

These higher d*N*/d*S* values can, in part, be explained by stronger positive selection acting on pollen-specific genes compared with sporophyte-specific genes, as indicated by a greater proportion of pollen-specific genes containing sites under positive selection (15.2% compared with 9.3%). However, an analysis of the DFEs of new nonsynonymous mutations revealed a higher frequency of effectively neutral mutations within pollen-specific genes. This indicates purifying selection is more relaxed within pollen-specific genes, suggesting the higher d*N*/d*S* rate within pollen-specific genes may have been caused by a greater proportion of slightly deleterious substitutions due to random drift.

### Polymorphism Levels Suggest Relaxed Selection on Pollen-Specific Genes

Polymorphism levels were significantly higher within pollen-specific genes. Both Watterson’s *θ* and *π* of nonsynonymous sites remained significantly higher within pollen-specific genes when controlling for expression and five further genomic differences (GC content, codon bias variance, gene length, average intron length, and gene density). In one of two recent studies, higher polymorphism rates were also found in pollen-specific genes for *A. thaliana* (Szövényi et al. 2013). In the second study, however, no difference was found between pollen-specific genes in general and random, nonreproductive genes in terms of nucleotide diversity (Gossmann et al. 2014), which, as discussed in the previous section, is possibly due to the more inclusive choice of genes in that study.

We also found significantly higher levels of putatively deleterious alleles (premature stop codons and frameshift mutations) within pollen-specific genes. This supports the conclusions of Szövényi et al. (2013) that the raised polymorphism levels indicate relaxed purifying selection on pollen-specific genes. In other words, comparatively weaker selective constraints are allowing deleterious alleles to accumulate at a greater rate within pollen-specific genes compared with those whose expression is restricted to the sporophyte.

### Has There Been a Recent Shift in Selection Strength?

The patterns in our data are compatible with a change in selection efficacy that is likely to have taken place since the speciation of *A. thaliana* and *A. lyrata*. The relatively recent switch from self-incompatibility to self-compatibility in *A. thaliana* (ca., 1 Ma; Tang et al. 2007) explains why we have observed evidence for relaxed purifying selection in polymorphism levels but stronger positive selection in divergence data for pollen-specific genes. The slightly higher proportion of adaptive substitutions (*α*) calculated for sporophyte genes with the DFE method is, due to this expected change in mating system and reduction in *N*_e_, thus unreliable, as previously discussed (Rousselle et al. 2018). The divergence data used to calculate d*N*/d*S* mainly represent a prolonged period of outcrossing (∼ 12 Ma), since the speciation of *A. thaliana* from *A. lyrata* occurred roughly 13 Ma (Beilstein et al. 2010). In contrast, the polymorphism data and frequencies of putative deleterious alleles reflect the recent selective effects of high selfing rates. This may also explain why more relaxed purifying selection was discovered for pollen-specific genes by the DoFE analysis, because it also relies on polymorphism data.

The evidence we have found for a more recent weaker selection on pollen-specific genes contrasts with findings for the outcrossing *Capsella grandiflora* (Arunkumar et al. 2013). In that study, the more efficient purifying and adaptive selection on pollen genes was linked to two possible factors: haploid expression and pollen competition. *A. thaliana* is a highly self-fertilizing species with selfing rates generally in the range of 95–99% (Platt et al. 2010), so haploid expression is unlikely to improve the efficacy of selection on pollen-specific genes relative to sporophyte genes. This is because most individuals found in natural populations are homozygous for the majority of loci, reducing the likelihood that deleterious alleles are masked in heterozygous state when expressed in a diploid tissue (Platt et al. 2010). A reduction in pollen competition can also be expected due to the probably limited number of pollen genotypes in highly selfing populations (Charlesworth D and Charlesworth B 1992; Mazer et al. 2010). However, outcrossing does occur in natural *A. thaliana* populations with one study reporting an effective outcrossing rate in one German population of 14.5% (Bomblies et al. 2010). Nevertheless, it appears that these generally rare outcrossing events may not be sufficient to prevent a reduction in pollen competition for *A. thaliana*.

So if we assume both masking and pollen competition are negligible forces when comparing selection on pollen-specific genes to sporophyte-specific genes, why is selection more relaxed on pollen-specific genes than sporophyte-specific genes rather than similar?

We have shown here that tissue specificity partly explains why selection is more relaxed on pollen genes. The full set of sporophyte-specific genes contains genes expressed across several tissues, and broadly expressed genes have been known to be under more efficient selection than tissue-specific genes due to their exposure to a higher number of selective constraints (Duret and Mouchiroud 2000; Liao et al. 2006; Slotte et al. 2011). Both pollen-specific genes and genes limited to one of three sporophytic tissues (xylem, guard cell, or root hair) showed raised levels of d*N*/d*S*, polymorphism, and frequency of deleterious mutations compared with broadly expressed sporophyte-specific genes (expressed in at least five tissues). Tissue specificity appeared to explain, to a certain extent, the reduced selection efficacy in pollen-specific genes as there was no longer a significant difference in polymorphism levels (*θ*_n_ and *π*_n_) or the frequency of frameshift mutations in pollen-specific genes compared with the tissue-specific, sporophytic genes (the frequency of stop codon mutations remained significantly higher). However, tissue specificity alone only partly explains the apparent, current more relaxed selection on pollen-specific genes. Once further genomic features (expression level, GC content, codon bias variance, gene length, average intron length, and gene density) were controlled for, all measures remained higher in pollen-specific genes even when compared with genes restricted to only one sporophytic tissue except for stop codon frequency.

The difference in rates of protein evolution between pollen-specific and sporophyte-specific genes could also be explained to a large extent by tissue specificity. As with polymorphism level and frequencies of deleterious mutations, d*N*/d*S* did not differ between pollen-specific and tissue-specific, sporophytic genes. However, d*N*/d*S* was significantly higher in both gene groups compared with broadly expressed, sporophytic genes. This suggests that the specificity of pollen genes to a small set of tissues is responsible for their elevated rates of protein evolution rather than their specific association with pollen tissues. Again, this only partially explains the raised d*N*/d*S* levels, as when controlling for differences in expression level and GC content, d*N*/d*S* remained significantly higher within pollen-specific genes.

Previously reported similar findings indicating relaxed purifying selection in pollen-specific genes in *A. thaliana* (Szövényi et al. 2013) were explained as possibly resulting from a combination of high tissue specificity and higher expression noise in pollen compared with sporophytic genes. However, the authors did not compare selection on pollen genes to tissue-specific sporophyte genes to isolate the effect of tissue specificity. We have shown here that tissue specificity does appear to play a role but does not alone explain the difference in selection strength between both groups of genes. Higher expression noise could then be an important factor influencing the level of deleterious alleles which exist for pollen genes in *A. thaliana*.

Expression noise has been found to reduce the efficacy of selection substantially and is expected to be considerably higher for haploid expressed genes (Wang and Zhang 2011). It is therefore likely that in the absence of pollen competition and the masking of deleterious sporophyte-specific genes, expression noise and high tissue specificity become dominant factors for pollen-specific genes of selfing plants. The loss of self-incompatibility in *A. thaliana* may therefore have led to a reduction in selection efficacy and the accumulation of deleterious alleles in pollen-specific genes.

## Conclusion

We have shown that, as in many other taxa, genes expressed in male reproductive tissues evolve at a quicker rate than somatic genes in *A. thaliana*. The greater divergence of pollen proteins to both *A. lyrata* and *C. rubella* compared with sporophytic genes can be attributed to stronger positive selection. However, intraspecific polymorphism data indicate a strong shift in this selection pattern may have occurred. Since the more recent loss of incompatibility in *A. thaliana*, selection appears to have become more relaxed in pollen-specific genes. This is likely due to a reduction in pollen competition and the masking of diploid, sporophytic genes as a result of high homozygosity levels. In outcrossing plants, haploid expression and pollen competition outweigh the negative impact of high tissue specificity and expression noise on the selection efficacy of pollen-specific genes. In the self-compatible *A. thaliana* high homozygosity has likely reduced the counteracting effects of pollen competition and haploid expression, leading to lower selection efficacy and an increased accumulation of deleterious mutations in pollen-specific compared with sporophyte-specific genes.

## Data Accessibility

All custom made scripts used in this study are deposited at the following dedicated GitHub site: https://github.com/MarkChristianHarrison/PloidyAndSelection.git.

## Supplementary Material

Supplementary data are available at *Genome Biology and Evolution* online.

## Supplementary Material

Supplementary_Matrial_evz127
