# Supplementary material for: Deleterious Mutation Accumulation in Arabidopsis thaliana Pollen Genes: A Role for a Recent Relaxation of Selection
Source: Genome Biol Evol. 2019 Jun 18;11(7):1939–51. doi: 10.1093/gbe/evz127 (PMC6640295; doi:10.1093/gbe/evz127)
Supplement: Supplementary_Matrial_evz127 [file supplementary_matrial_evz127.pdf]

Supplementary Material

Supplementary figures

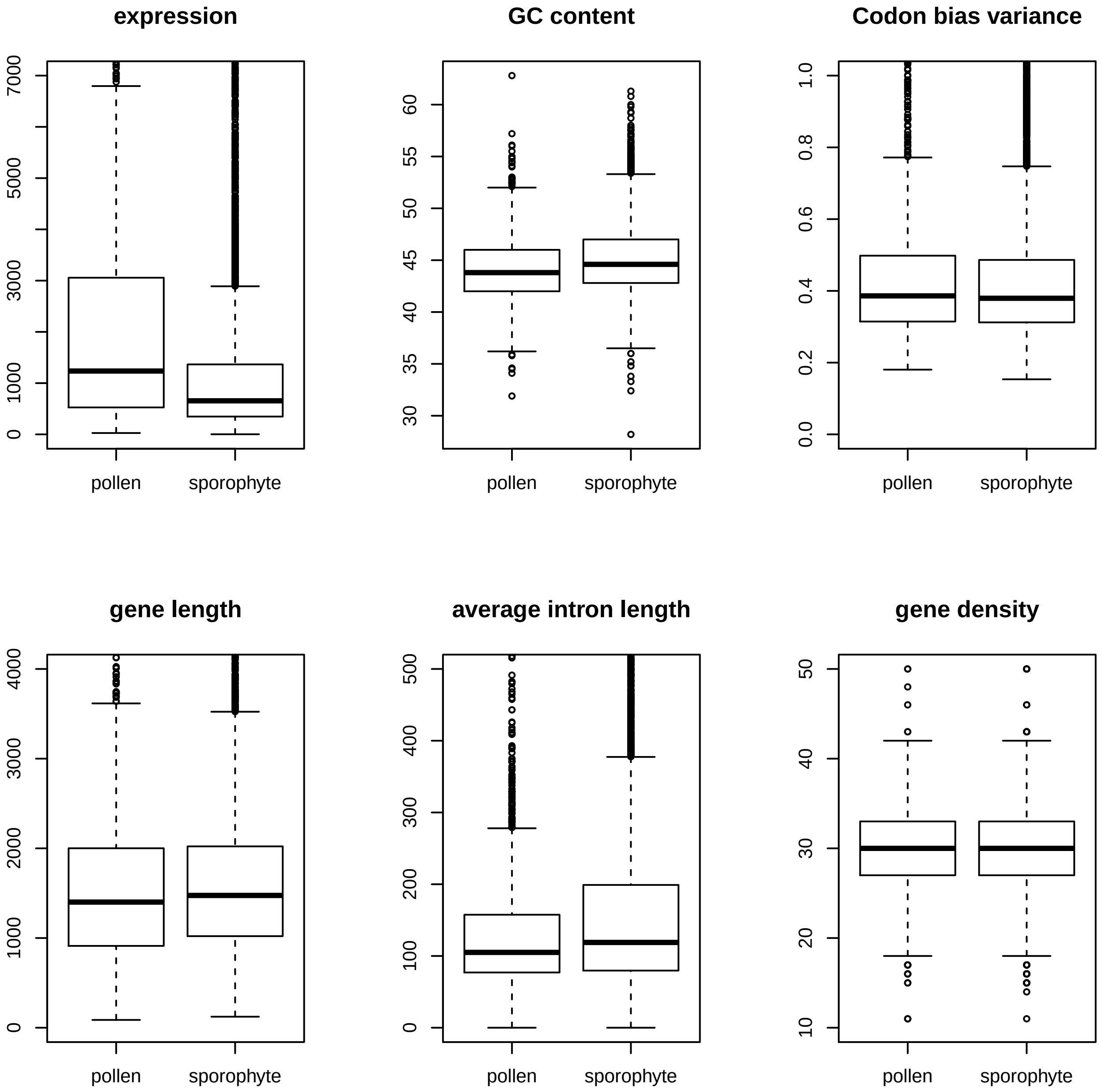

Figure S1: Genomic features of pollen- and sporophyte specific genes.

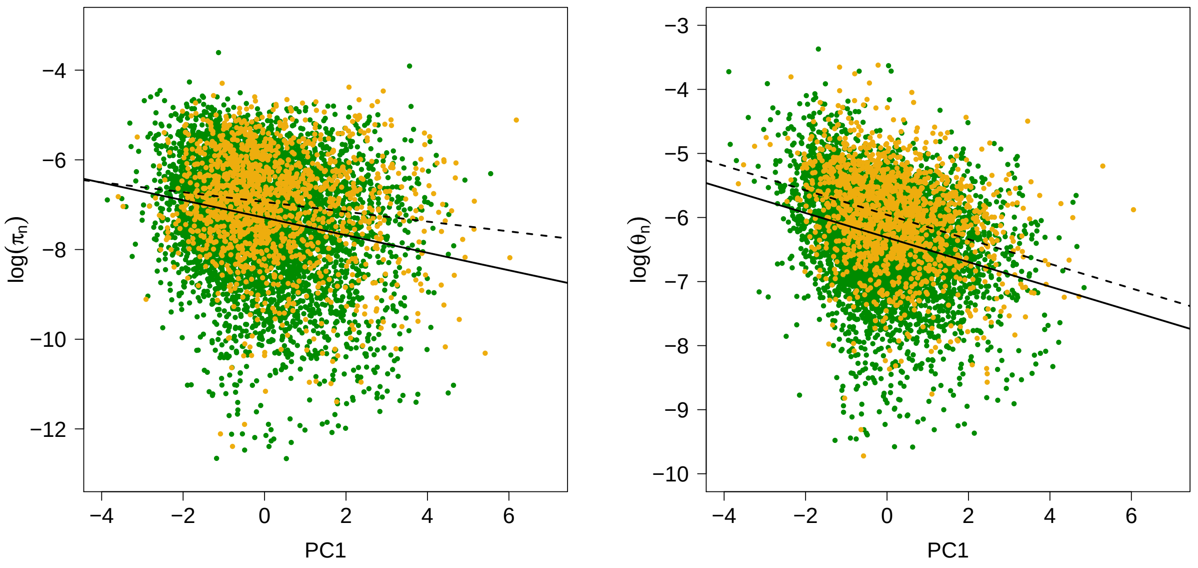

(a) (b)

Figure S2: ANCOVA analysis with PC1 (6 genomic variables) as continuous variable reveals both higher π_n (a) and higher θ_n (b) among pollen-specific (dark grey points and dashed line) than sporophyte-specific genes (light grey points and solid line).

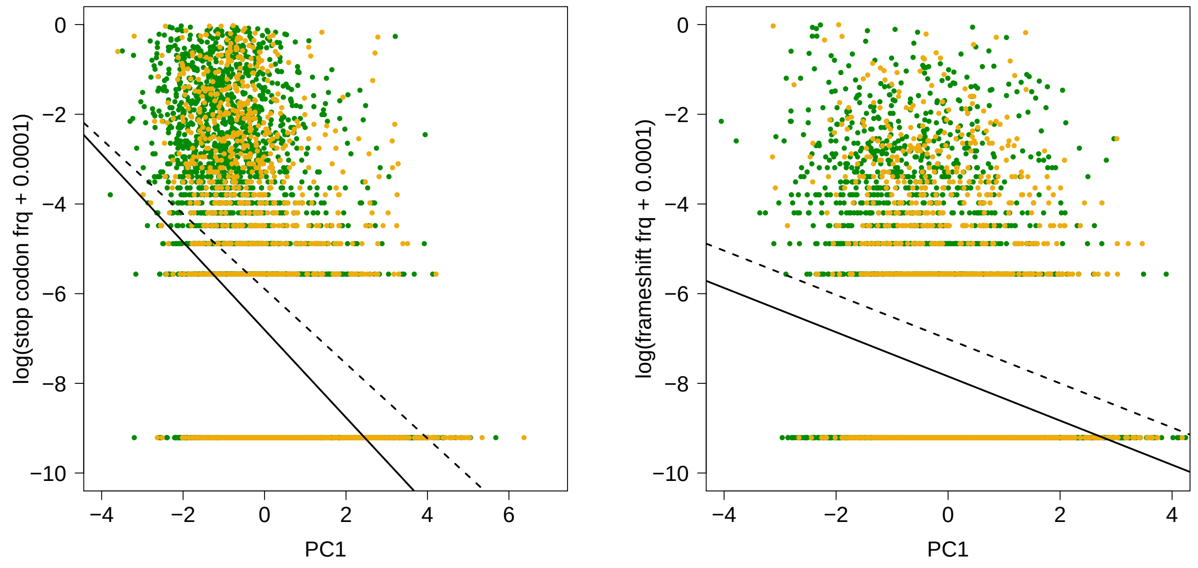

(a) (b)

Figure S3: ANCOVA analysis with PC1 (6 genomic variables) as continuous variable reveals significantly higher frequency of stop codon mutations (a) and frameshift mutations (b) among pollen-specific (dark grey points and dashed line) than sporophyte-specific genes (light grey points and solid line).

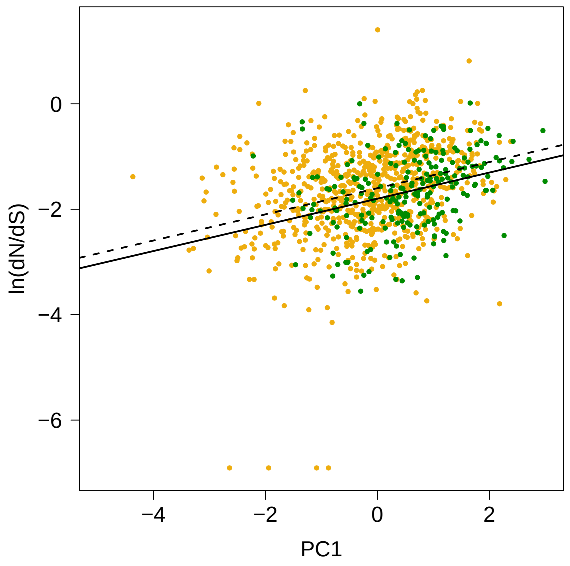

Figure S4: ANCOVA analysis of dN/dS within pollen-specific (yellow points and dashed line) and tissue specific, sporophyte genes (green points and solid line) with PC1 (expression and GC content) as the continuous variable .

figure 10

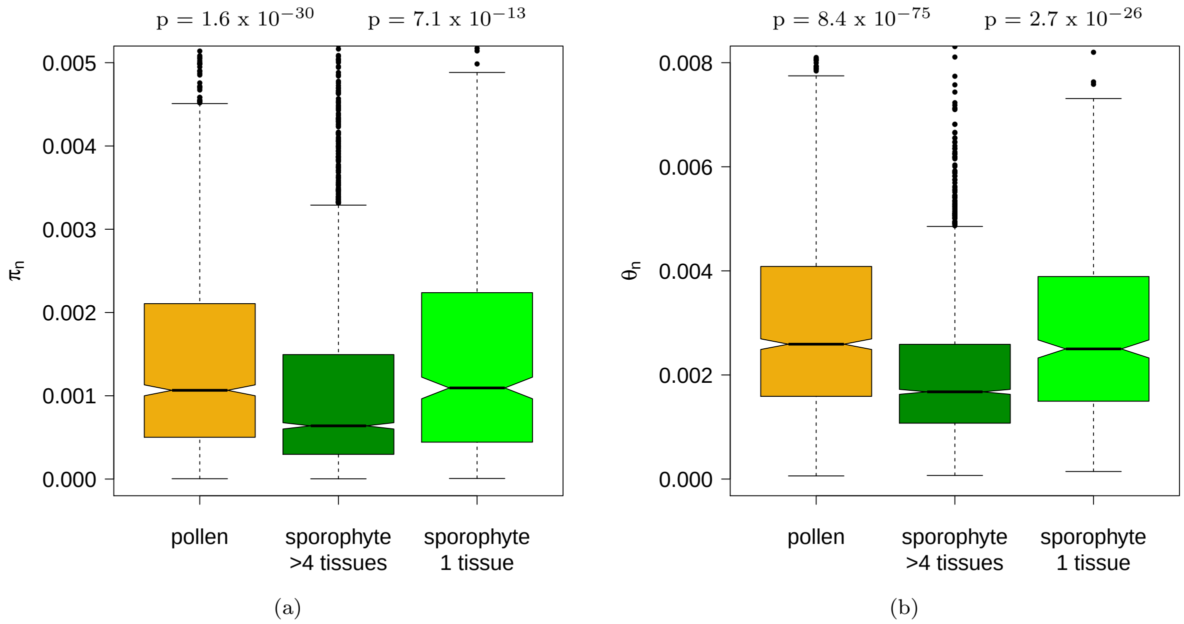

Figure S5: Non-synonymous nucleotide diversity (a) and non-synonymous Watterson's theta (b) within pollen-specific genes, broadly expressed sporophyte-specific genes and genes specific to guard cells, xylem or root hair.

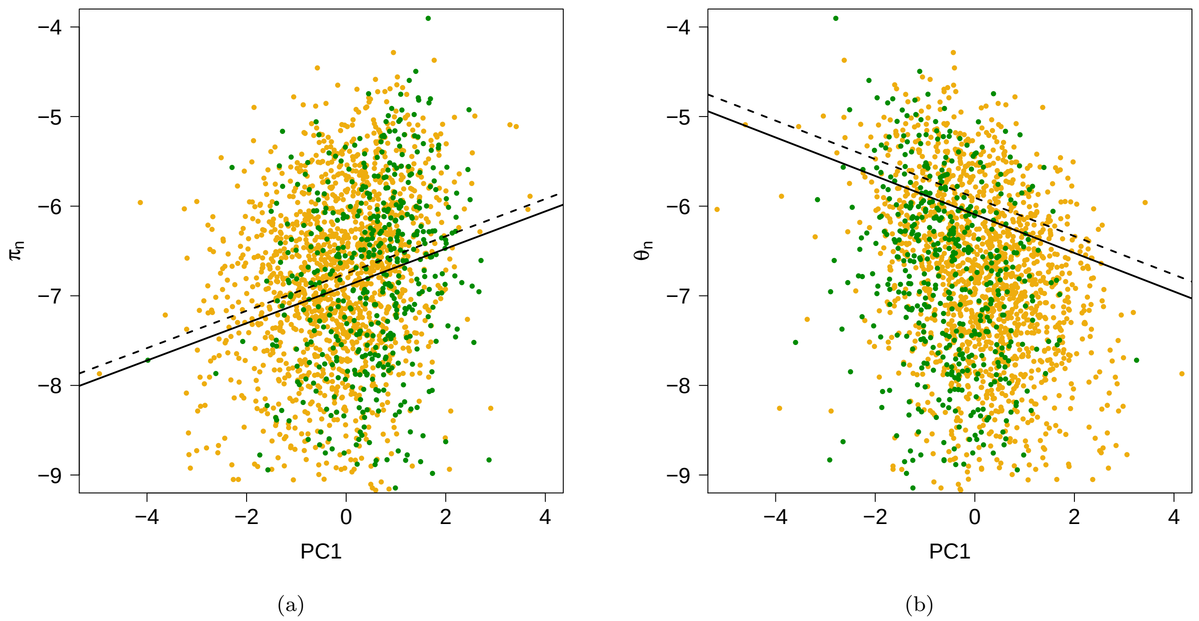

Figure S6: ANCOVAS comparing π_n (a) and θ_n (b) within pollen-limited genes (yellow points and dashed line) to tissue-specific, sporophytic genes (green points and solid line) while controlling for the first PC of a PCR.

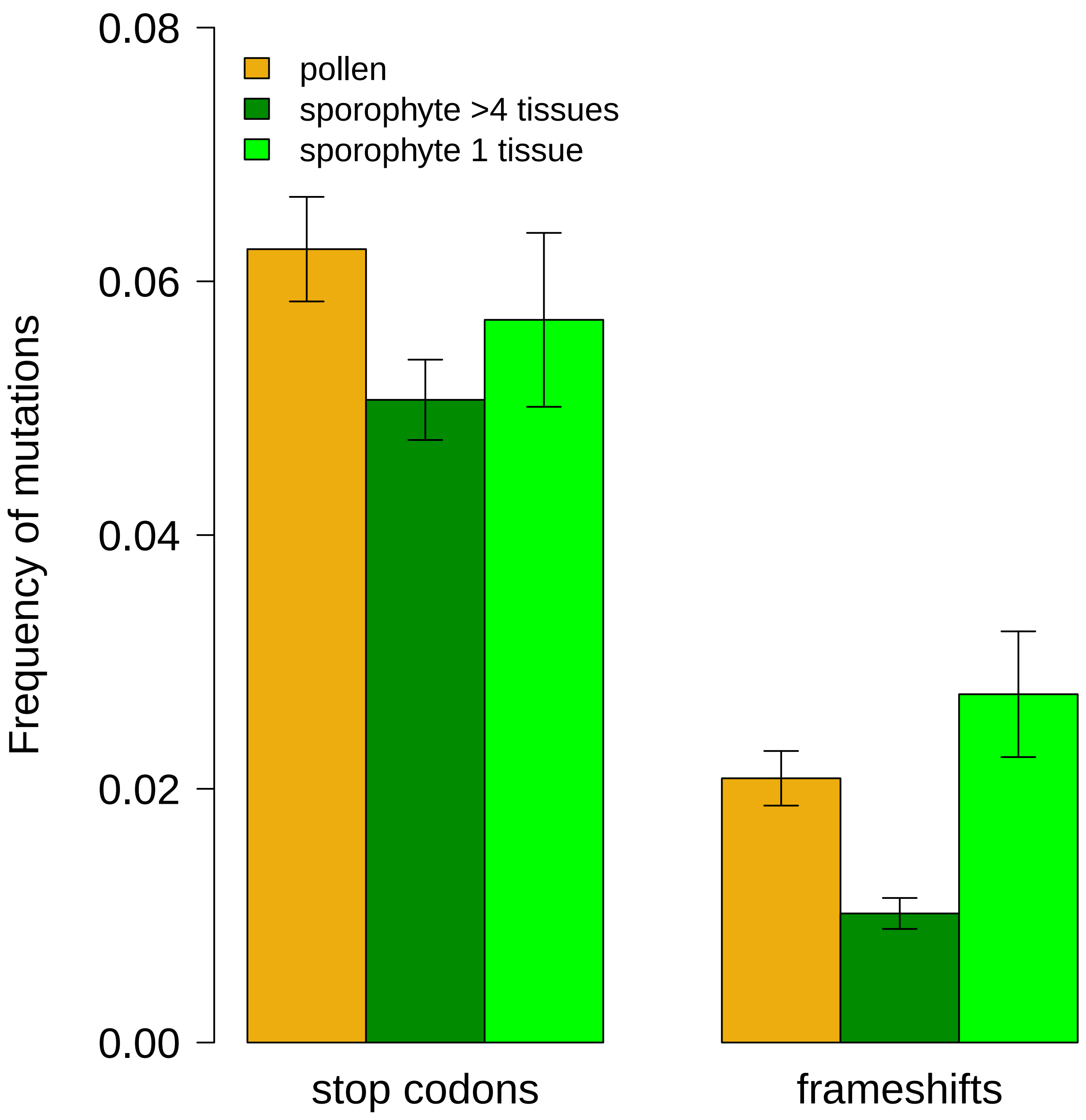

Figure S7: Frequency of stop codon and frameshift mutations within pollen-specific genes, broadly expressed sporophytic genes (at least 5 tissues) and tissue specific genes (expression restricted to guard cell, xylem or root hair tissues). Shown are means and standard error.

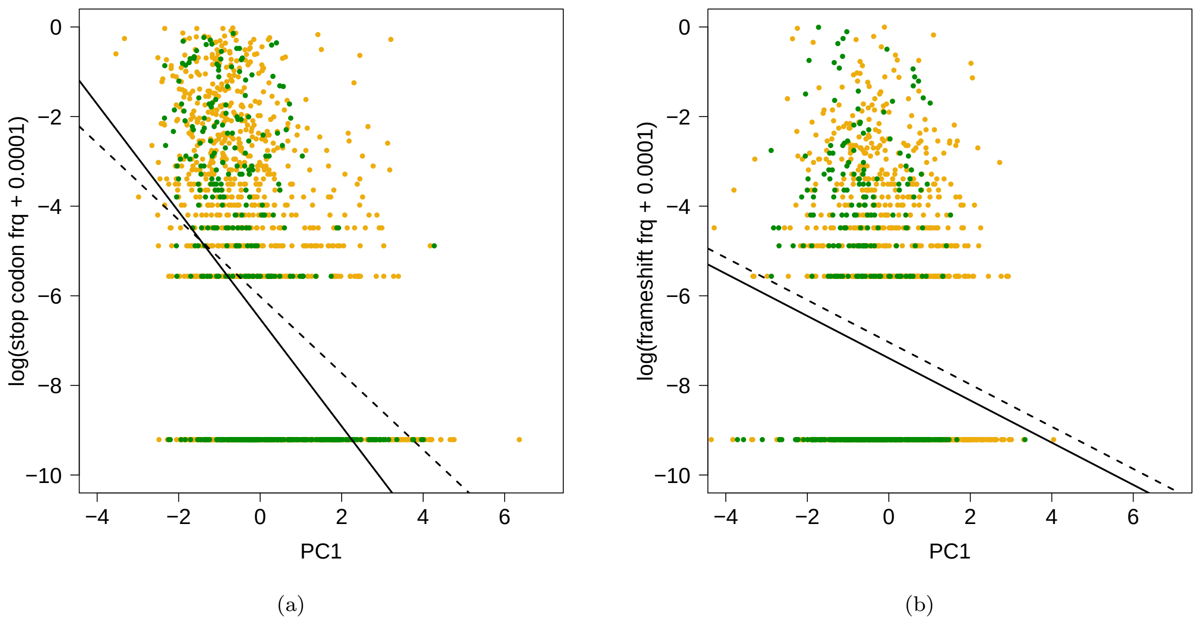

Figure S8: ANCOVAS comparing the frequency of stop codon mutations (a) and frameshift mutations (b) within pollen-limited genes (yellow points and dashed line) to tissue-specific, sporophytic genes (green points and solid line) while controlling for the first PC of a PCR.

Table S1: Expression data sets.

	Dataset	Description	Chips	Original source
Haploid	UNM	Uninucleate microspore	2	Honys & Twell, 2004
	BCP	Bicellular pollen	2	Honys & Twell, 2004
	TCP	Tricellular Pollen	2	Honys & Twell, 2004
	MPG	Mature Pollen	2	Honys & Twell, 2004
	GP*	Pollen Tube Grouped	6	Qin et al., 2009 ; Wang et al., 2008
	PT4*	Pollen Tube Grouped	6	Qin et al., 2009 ; Wang et al., 2008
	SPC	Sperm Cell	3	Borges et al., 2008
Diploid	SL	Silique	30	NASC
	LF	Leaves	36	NASC
	GC	Guard Cell	3	NASC
	PT**	Petiole	3	NASC
	ST	Stems	2	NASC
	HP	Hypocotyl	8	NASC
	XL	Xylem	3	NASC
	CR	Cork	3	NASC
	RT	Roots	11	NASC
	RH	Root hair elongation zone	3	NASC

NASC: Nottingham Arabidopsis Stock Centre.
* GP and PT4 were combined to one data set called PT, selecting the highest expression level of the two for each gene.
** Renamed PET

Table S2: Comparison of chromosomal positions of pollen and sporophyte genes. Mann Whitney U test.

Chromosome	W	p
1	2.79×10^5	0.137
2	1.00×10^5	0.071
3	1.72×10^5	0.315
4	8.54×10^4	0.267
5	2.31×10^5	0.241

Table S3: dN/dS between *A. thaliana*, *A. lyrata* and *C. rubella*. Values are means (and medians); p-values denote significance of difference in dN/dS betweeen pollen and sporophyte genes on each branch; significance was tested with Mann Whitney U test; p-values are Bonferroni corrected for multiple testing.

	Pollen	Sporophyte	p value
A. thaliana vs. *A. lyrata*	0.2689 (0.2106)	0.1963 (0.1664)	4.3×10^{-24}
A. thaliana vs. *C. rubella*	0.2409 (0.2036)	0.1801 (0.1567)	8.8×10^{-22}
A. lyrata vs. *C. rubella*	0.2370 (0.1945)	0.1818 (0.1568)	1.3×10^{-15}

Table S4: Nonsynonymous pi within 5 equal bins along the PC1 axis. Shown are medians (means).

	< 20%		20% - 40%		40% - 60%		60% - 80%		> 80%	
Pollen	1.0×10^{-3}	(1.7×10^{-3})	1.1×10^{-3}	(1.7×10^{-3})	1.1×10^{-3}	(1.7×10^{-3})	1.1×10^{-3}	(1.6×10^{-3})	8.4×10^{-4}	(1.5×10^{-3})
Sporophyte	1.0×10^{-3}	(1.7×10^{-3})	8.6×10^{-4}	(1.4×10^{-3})	7.2×10^{-4}	(1.2×10^{-3})	6.7×10^{-4}	(1.1×10^{-3})	6.0×10^{-4}	(1.0×10^{-3})
p	ns		1.1×10^{-3}		1.1×10^{-8}		5.8×10^{-6}		1.0×10^{-5}	

Table S5: Frequency of stop codons within 5 equal bins along the PC1 axis. Shown are medians (means).

	< 20%		20% - 40%		40% - 60%		60% - 80%		> 80%	
Pollen	0.028	(0.113)	0.011	(0.111)	0.004	(0.056)	0	(0.033)	0	(0.015)
Sporophyte	0.015	(0.106)	0.004	(0.063)	0	(0.045)	0	(0.022)	0	(0.006)
p	non significant		5.7×10^{-6}		1.1×10^{-5}		6.3×10^{-8}		8.3×10^{-11}	

Table S6: Differences in 6 genomic variables between pollen-specific genes and genes limited to one of three sporophytic tissues. Values are means ± standard error of the mean; significance was tested with Mann Whitney U test; p-values are Bonferroni corrected for multiple testing.

	Pollen-specific genes			guard cell, xylem or root hair		p
Expression level	2,562.30	± 86.49	>	446.24	± 26.82	1.0×10^{-77}
GC content (%)	44.20	± 0.08	<	44.80	± 0.17	4.5×10^{-3}
Codon bias variance	0.46	± 0.01	>	0.39	± 0.01	2.2×10^{-6}
gene length	1,570.30	± 24.41	=	1,561.71	± 36.20	not significant
average intron length	124.44	± 3.23	=	152.49	± 9.14	not significant
gene density (per 100kb)	29.99	± 0.12	=	29.48	± 0.30	not significant
